# Mechanisms Underlying the Regulation of HP1γ by the NGF-PKA Signaling Pathway

**DOI:** 10.1038/s41598-018-33475-y

**Published:** 2018-10-10

**Authors:** Seungmae Seo, Angela Mathison, Adrienne Grzenda, Jewel Podratz, Ezequiel Calvo, Stephen Brimijoin, Anthony Windebank, Juan Iovanna, Gwen Lomberk, Raul Urrutia

**Affiliations:** 1 Lieber Institute for Brain Development, Baltimore, MD USA; 2 Genomic Sciences and Precision Medicine Center (GSPMC), Medical College of Wisconsin, Milwaukee, WI USA; 3 University of California, Los Angeles, Psychiatry Residency Program, Los Angeles, CA USA; 4 Department of Neuroscience, Mayo Clinic, Rochester, MN USA; 5 Centre Génomique du Centre de Recherche du CHUL Research Center, Ville de Québec, Quebec, Canada; 6 Department of Pharmacology and Experimental Therapeutics, Mayo Clinic, Rochester, MN USA; 7 Centre de Recherche en Cancérologie de Marseille (CRCM), INSERM U1068, CNRS UMR 7258, Institut Paoli-Calmettes, Aix Marseille Université, Marseille, France; 8 Division of Research, Department of Surgery, Medical College of Wisconsin, Milwaukee, WI USA; 9 Department of Pharmacology and Toxicology, Medical College of Wisconsin, Milwaukee, WI USA

## Abstract

Heterochromatin protein 1 γ (HP1γ) is a well-known chromatin protein, which regulates gene silencing during the execution of processes associated with embryogenesis, organ maturation, and cell differentiation. We find that, *in vivo*, the levels of HP1γ are downregulated during nervous system development. Similar results are recapitulated *in vitro* during nerve growth factor (NGF)-induced neuronal cell differentiation in PC12 cells. Mechanistically, our experiments demonstrate that in differentiating PC12 cells, NGF treatment decreases the association of HP1γ to silent heterochromatin, leads to phosphorylation of this protein at S83 via protein kinase A (PKA), and ultimately results in its degradation. Genome-wide experiments, using gain-of-function (overexpression) and loss-of-function (RNAi) paradigms, demonstrate that changing the level of HP1γ impacts on PC12 differentiation, at least in part, through gene networks involved in this process. Hence, inactivation of HP1γ by different post-translational mechanisms, including reduced heterochromatin association, phosphorylation, and degradation, is necessary for neuronal cell differentiation to occur. Indeed, we show that the increase of HP1γ levels has the reverse effect, namely antagonizing neuronal cell differentiation, supporting that this protein acts as a barrier for this process. Thus, these results describe the regulation and participation of HP1γ in a novel membrane-to-nucleus pathway, through NGF-PKA signaling, which is involved in NGF-induced neuronal cell differentiation.

## Introduction

Similar to members of the Polycomb-group complexes, HP1 was first described in Drosophila as a suppressor of position-effect variegation, a heterochromatin-mediated process for silencing gene expression^1^. In the human genome, HP1α/CBX5, HP1β/CBX1, and HP1γ/CBX3 are located on chromosomes 12q13.13, 17q21.32 and 7p15.2, respectively^2^. All three HP1 proteins are comprised of a highly conserved chromodomain at the amino terminus, which binds to trimethyl histone H3 lysine 9 (H3K9me3), and a chromoshadow domain at the carboxy terminus that enables protein-protein interactions with other epigenetic regulators including HP1 itself. Due to the similarities in these domains and their ability to dimerize with each other, HP1 isoforms have functional redundancy^3^. In addition to the redundant functions, the highly variant linker domain, connecting the two conserved globular domains, enables each isoform to also have divergent functionality. Several studies have shown that localization patterns in the nucleus, post-translational modifications, and tissue-specific phenotypes in knockout mice differ among the three HP1 isoforms^4–9^.

Previously, we have demonstrated that HP1 can form complexes with sequence-specific transcription factors (e.g. KLF11), which are involved in the regulation of neuron specific genes, such as the Dopamine receptor D2^10^. Other groups have implicated a role of HP1γ in neuronal differentiation^11,12^. However, the mechanism by which HP1γ regulates neuronal cell differentiation and cell functions remains to be established. In this study, we used well-established cell models for studying NGF-mediated neuronal differentiation in combination with the transduction of genetic mutants, signaling experiments, and genome-wide transcriptional profiling. Combined, these approaches allow us to provide evidence supporting the notion that the timely regulation of the intracellular concentration and post-translational modification of HP1γ is necessary for proper differentiation that occurs through an NGF-PKA-HP1γ pathway.

## Results

### HP1γ Levels Decrease During Nervous System Development

**Figure 1:**
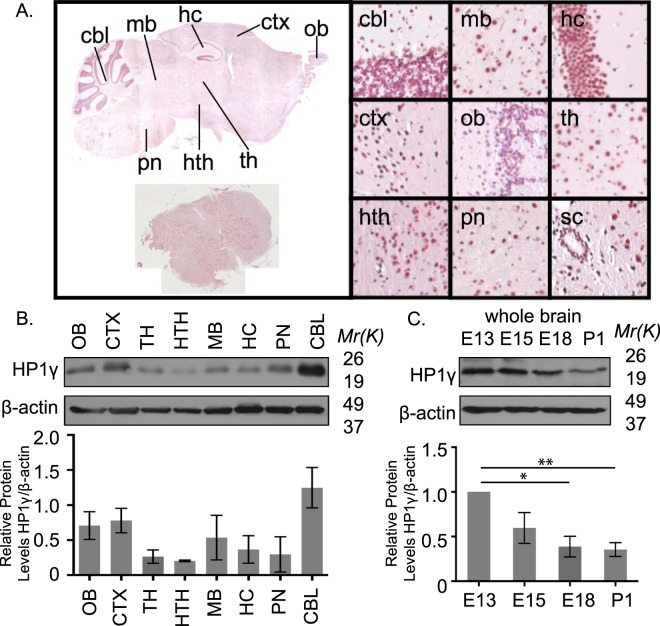
HP1γ levels vary in adult and developing mouse brain. (**A**) HP1γ staining in mouse brain sections shows robust expression in nuclei. (**B**) HP1γ protein levels were measured in dissected mouse brain areas. Representative western blot (top) and quantification (bottom) are shown. (ob: olfactory bulb, ctx: cortex, th: thalamus, hth: hypothalamus, mb: mid brain, hc: hippocampus, pn:pons, cbl: cerebellum). Full-length blots are presented in Supplementary Fig. 1A. (**C**) Representative western blot (top) and quantification (bottom) show decreasing levels of HP1γ in mouse whole brain samples from embryonic (**E**) days 13, 15, 18 and post-natal (P) day 1. Full-length blots are presented in Supplementary Fig. 1B. All protein level quantifications are normalized to corresponding β-actin levels. Error bars represent standard error. (*p < 0.05, **p < 0.01).

Since the expression and function of HP1γ in the nervous system has not been extensively investigated, we initiated our study by performing immunohistochemistry against this protein in brain tissues from 6-week-old C57BL/6 mice. These experiments demonstrate that HP1γ is readily detected in the nucleus of most neurons in the brain (Fig. 1A). We therefore measured HP1γ protein levels by western blot of samples isolated from the same brain areas in 6-week-old mice. We found that the expression levels of HP1γ are highest in the cerebellum, followed by cortex and olfactory bulb, while hypothalamus and thalamus showed the lowest expression levels, regardless of animal gender (Fig. 1B; Supplementary Fig. 1A). Since several members of the HP1 family of proteins have been shown to play a role in developmental pathways, we subsequently measured HP1γ expression in the developing brain from mouse pups at embryonic days (E) 13, 15, 18 and post-natal day (P) 1. We found that HP1γ levels significantly decreased during maturation of whole embryonic brain until the time of birth (Fig. 1C; E18; 38.8 ± 8.2%, p < 0.05; P1; 35.4 ± 5.5%, p < 0.01 compared to E13; and Supplementary Fig. 1B). Similarly, analysis of the BrainSpan dataset^13^, which provides transcriptome-wide measurements of human brain from 8 weeks postconception through 40 years of age, showed a decrease in HP1γ (CBX3) RNA levels during gestation (Supplementary Fig. 2A). Furthermore, inquiry of the Cortecon dataset^14^, representing iPSC derived neuronal differentiation, also demonstrated a decrease in HP1γ (CBX3) RNA levels during the progression of differentiation (Supplementary Fig. 2B). Combined, these results demonstrate that HP1γ protein is widely distributed in the mouse nervous system and undergoes overall downregulation during development of the nervous system, suggesting a role for this protein in neuronal cell maturation.

### Downregulation of HP1γ Occurs During Serum and NGF-Induced Neuronal Cell Differentiation

**Figure 2:**
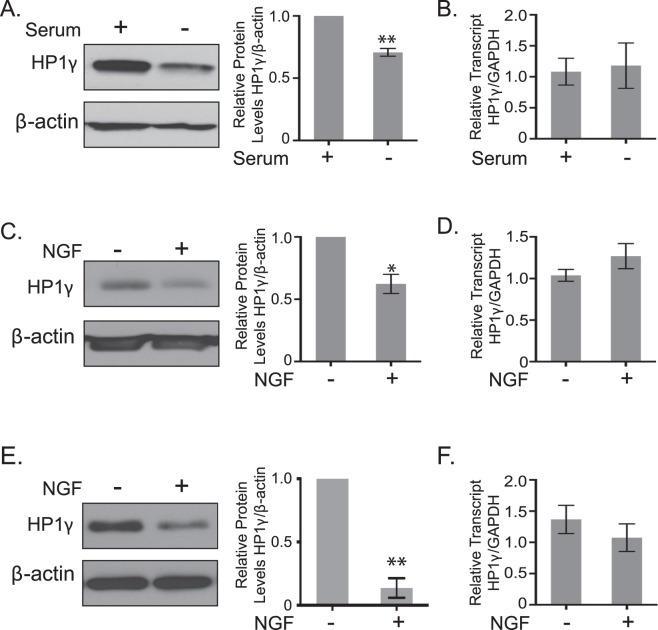
Total HP1γ levels decrease in response to differentiation of neuronal cell cultures. (**A**) Western blot and quantification show significant reduction of HP1γ protein levels in serum starved N1E115 cell line compared to control conditions. β-actin is used as a loading control. Full-length blots are presented in Supplementary Fig. 3A. (**B**) qPCR results indicate that *HP1*γ mRNA levels in N1E115 cells did not change with serum starvation. *GAPDH* is used as a housekeeping control among samples. (**C**) Western blot demonstrates a decrease of HP1γ protein levels in PC12 cells differentiated with 72 hours of 100 ng/ml NGF treatment. Quantification is shown relative to the loading control, β-actin and normalized to control conditions. Full-length blots are presented in Supplementary Fig. 3B. (**D**) Levels of *HP1*γ mRNA in PC12 cells did not change significantly with NGF induced differentiation, as measured by qPCR and normalized to a housekeeping gene, *GAPDH*. (**E**) Western blot of lysates from whole DRGs of E13 mice (−NGF) and treated with 10 ng/ml NGF for 48 hours (+NGF) shows a decrease of HP1γ protein levels upon differentiation. β-actin is used as a loading control. Full-length blots are presented in Supplementary Fig. 3C. (**F**) mRNA levels of *HP1*γ in DRG did not change significantly with NGF induced differentiation. *GAPDH* is used as a housekeeping control among samples. Error bars represent standard error. (*p < 0.05, **p < 0.01).

Interestingly, a recent study demonstrated that HP1γ protein levels increase in cortical neurons of the mouse embryonic neocortex^11^. Using immunohistochemistry, this study specifically compared the cortical neurons before and after their migration from the ventricular zone. The whole brain homogenates used in our study (Fig. 1C) also contained non-neuronal cells and therefore did not exclusively represent neuronal cell differentiation. Thus, to further investigate how HP1γ levels are regulated during neuronal cell differentiation, we examined two cell lines that are widely used to study this process; PC12 and N1E115. We first examined HP1γ at the protein and mRNA levels in N1E115, a well-established model for studying differentiation of adrenergic neurons^15^, before and after differentiation by serum starvation. Congruent with the idea that HP1γ is downregulated during CNS maturation, Fig. 2A shows that HP1γ protein levels in N1E115 is reduced with differentiation (70.7 ± 1.8%, p < 0.01; Supplementary Fig. 3A). In contrast, HP1γ mRNA levels (Fig. 2B) did not change with differentiation, suggesting that its downregulation occurs at a post-transcriptional level. Similar results were observed during NGF-induced PC12 cell differentiation. Lysates from PC12 cells differentiated with 100 ng/mL NGF for 72 hours also demonstrated that HP1γ levels decreased with PC12 cell differentiation (71.9 ± 8.1% of control p < 0.05; Fig. 2C; Supplementary Fig. 3B), while its corresponding mRNA levels, as measured by qPCR, did not change (Fig. 2D). Similarly, in *ex vivo* dorsal root ganglion (DRG) cultures, NGF treatment induced neural differentiation, and neurite outgrowth correlated with decreased levels of HP1γ protein (13.5 ± 7.7% of control p < 0.01; Fig. 2E; Supplementary Fig. 3C) without a change at the transcript level (Fig. 2F). Therefore, we subsequently considered the role that signaling of the NGF pathway plays in the dynamics of this protein as it relates to its nuclear functions.

### The Localization of HP1γ Changes in Response to the NGF Pathway

**Figure 3:**
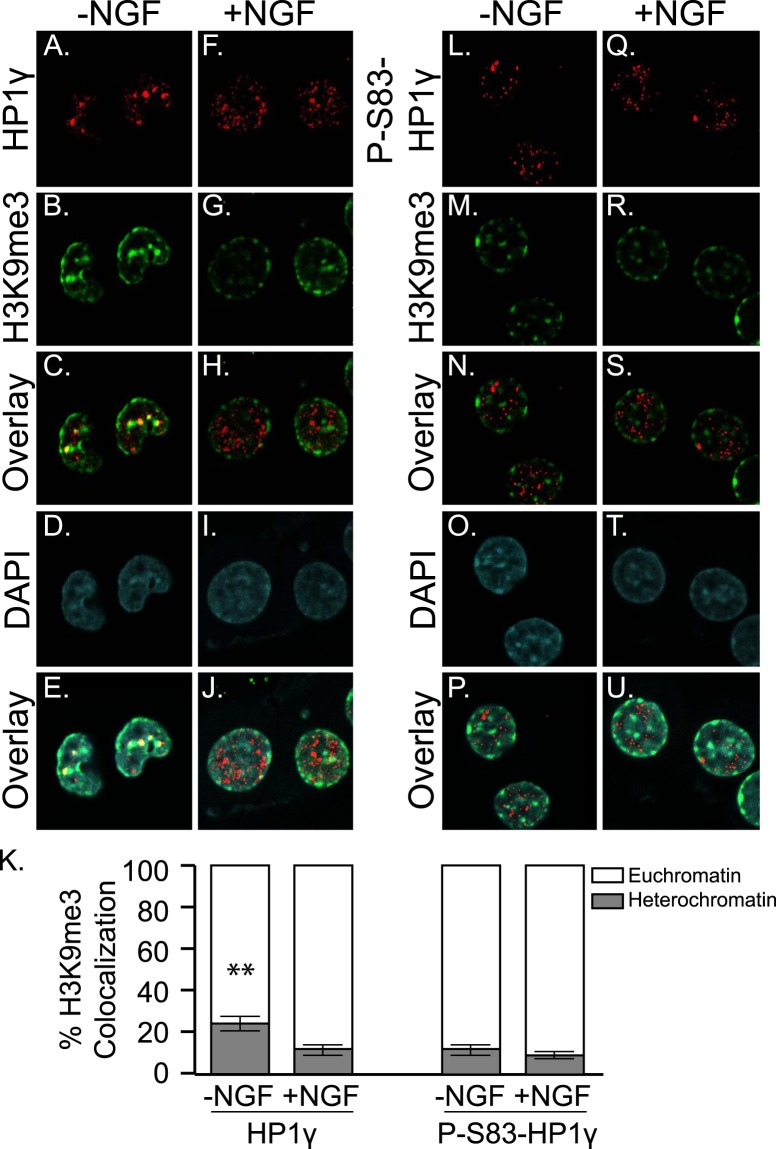
NGF results in relocalization of HP1γ. (**A**–**J**) Representative images for HP1γ (red) in PC12 cells without (**A**) and with 4 hours of 100 ng/ml NGF treatment (**F**). H3K9me3 (green; **B**,**G**) is used to mark heterochromatin. Overlay images indicate areas of heterochromatic localization in H3K9me3-rich regions and euchromatic localization in H3K9me3-poor regions (**C**,**H**). DAPI staining marks nuclei (**D**,**I**) and overlay of all 3 channels is also shown (**E**,**J**). (**K**) Co-localization of HP1γ and H3K9me3 was quantified at the pixel level. Error bars represent standard error. (**p < 0.01) (**L**–**U**). Representative images for P-Ser83-HP1γ (red) and H3K9me3 (green) under the same conditions with overlay and DAPI images as shown for total HP1γ. Quantification of euchromatic versus heterochromatic signal for P-Ser83-HP1γ at the pixel level is also shown in K.

Signaling pathways, such as NGF, frequently result in the transmission of signals from the cellular membrane to the nucleus. As part of this cellular response, re-localization of some effector proteins is often induced. Therefore, we investigated the sub-nuclear localization of HP1γ in response to NGF. First, we established the localization of total HP1γ in PC12 cells under proliferating (no NGF treatment) conditions. Using immunofluorescence (IF) staining, we compared the localization of HP1γ (red, Fig. 3A) to that of the H3K9me3 mark (green, Fig. 3B; overlay Fig. 3C), which is known to mark heterochromatic regions. Cells were counterstained with DAPI (blue, Fig. 3D) to visualize the nucleus with light euchromatic and intense heterochromatic regions. HP1γ was exclusively localized to the nucleus with no significant staining in the cytoplasm. Furthermore, nuclear HP1γ staining was in both, euchromatic and heterochromatic regions, as evidenced by signal overlay with H3K9me3 (Fig. 3C,E). Upon differentiating conditions induced with 4 hours of NGF treatment (100 ng/ml), localization of total HP1γ became more euchromatic (Fig. 3F–J). Pixel analysis of at least 200 cells per condition was performed to evaluate this change in localization of total HP1γ (Fig. 3K). Under proliferating conditions (-NGF), 24.5 ± 3.0% of total HP1γ was in heterochromatin (75.5 ± 3.0% in euchromatin). When the PC12 cells were differentiated with NGF, only 11.7 ± 2.0% of total HP1γ was in heterochromatin (88.3 ± 2.0% in euchromatin), which was a significant change in localization (p < 0.01). Previous studies demonstrated that in other cell lines, HP1γ becomes exclusively localized to euchromatic regions of the nucleus upon Ser83 phosphorylation^16,17^. Therefore, we investigated if the localization pattern of total HP1γ after NGF treatment resembled the localization of phosphorylated HP1γ-Ser83 (Fig. 3L–U). Indeed, the localization patterns of P-Ser83-HP1γ (red, Fig. 3Q,S) and total HP1γ (red, Fig. 3F,H) during NGF-induced differentiation were similar, suggesting that the change of localization observed with total HP1γ after NGF treatment may indicate phosphorylation of this protein. The phosphorylated population of HP1γ is specifically localized to euchromatin, and therefore, in an expected manner, the phosphorylation-specific antibody did not detect a significant change in the proportion of P-Ser83-HP1γ in euchromatin versus heterochromatin between no treatment and with NGF treatment (Fig. 3K; 11.9 ± 2.5% in heterochromatin and 88.1 ± 2.5% in euchromatin without NGF; Fig. 3L–P versus 9.5 ± 1.4% in heterochromatin and 90.5 ± 1.4% in euchromatin with NGF; Fig. 3Q–U). Together, these immunofluorescence data on HP1 localization in response to NGF treatment demonstrate that re-localization of HP1γ occurs within the nucleus, associating with localization of P-Ser83-HP1γ, which marks the inactivation of the repressive transcriptional function of this protein. These results prompted us to investigate whether NGF mediates phosphorylation of this chromatin protein.

### The NGF-PKA Pathway Induces Phosphorylation of HP1γ Early in NGF-induced PC12 Differentiation

**Figure 4:**
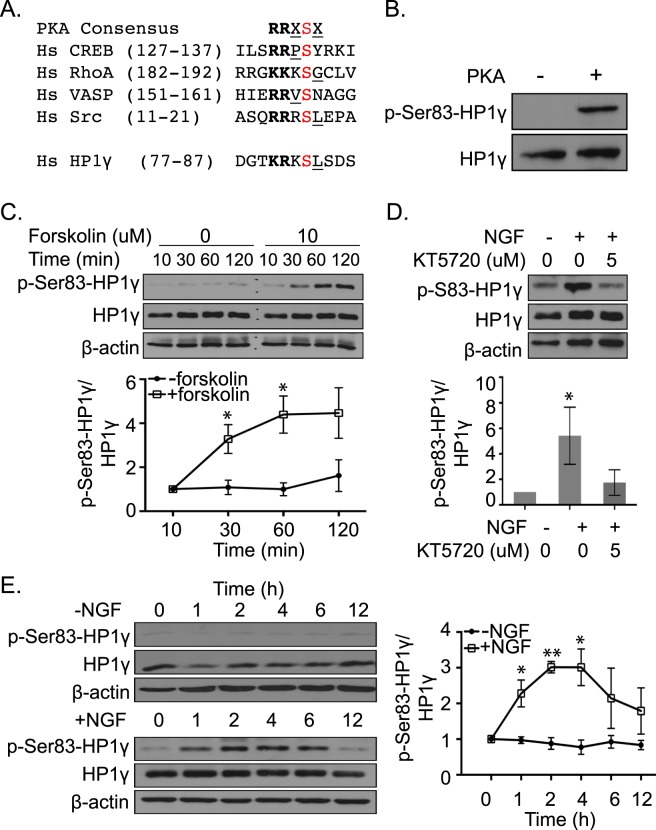
Phosphorylation of the HP1γ Ser83 residue occurs early in NGF-induced differentiation of PC12 cells. (**A**) Comparison between the HP1γ phosphorylation site and consensus sequence for other proteins phosphorylated in response to the NGF-PKA pathway predicts HP1γ Ser83 to be a PKA target. Amino acid positions are denoted in parenthesis. PKA phosphorylation sites are indicated in red. Positively charged and hydrophobic residues within the PKA consensus are in bold and underlined, respectively. (**B**) Purified HP1γ incubated with PKA *in vitro* phosphorylates HP1γ at the Ser83 residue. Total HP1γ is used as the loading control. Full-length blots are presented in Supplementary Fig. 4A. (**C**) Western blot (top) and quantification (bottom) show total HP1γ and P-Ser83-HP1γ levels in PC12 after forskolin (10 μM) treatment. Full-length blots are presented in Supplementary Fig. 4B. (**D**) Western blot (top) and quantification (bottom) show induction of P-Ser83-HP1γ by NGF is inhibited by KT5720 (5 μM) treatment. Full-length blots are presented in Supplementary Fig. 4C. (**E**) Western blot shows the levels of P-Ser83-HP1γ and total HP1γ in PC12 cells over time, with or without 100 ng/ml NGF treatment. P-Ser83-HP1γ is transiently induced during differentiation. Full-length blots are presented in Supplementary Fig. 5. β-actin is used as the loading control (**C**–**E**). Error bars represent standard error. (*p < 0.05, **p < 0.01).

Using the cervical cancer cell line, HeLa, we have previously reported that Ser83-HP1γ can be phosphorylated by PKA in interphase, which relocates the protein to euchromatic regions^16^. Additional studies have reported that phosphorylation of HP1γ at Ser83 regulates cell proliferation, differentiation, and senescence in non-neuronal cell populations^16–20^. However, whether this phosphorylation event occurs in neurons and its downstream consequences have not been studied. Eukaryotic Linear Motif analysis of the residues surrounding HP1γ-Ser83 match the consensus sequence for PKA (Fig. 4A), an important mediator of NGF signal^21^, similar to other proteins phosphorylated in response to the NGF-PKA pathway, such as CREB, RhoA, VASP and Src^21–24^. In *vitro* kinase assays not only confirmed that PKA phosphorylates HP1γ-Ser83 *in vitro* (Fig. 4B; Supplementary Fig. 4A), but also the ability of the phospho-specific antibody to detect this signal^16^. In PC12 cells, we observed that even in the absence of NGF, PKA activation by forskolin (10 µM for 30 minutes) significantly increased the levels of P-Ser83-HP1γ (Fig. 4C, p-value < 0.05; Supplementary Fig. 4B). Conversely, PKA inhibition by pre-treatment with KT5720 (5 µM) abolished the NGF-induced increase of P-Ser83-HP1γ (Fig. 4D; Supplementary Fig. 4C). We evaluated the ratio of P-Ser83-HP1γ levels to total HP1γ protein upon NGF treatment at time points that preceded its degradation observed after 72 hours of treatment (Fig. 2C). While total HP1γ levels remained relatively constant, levels of P-Ser83-HP1γ significantly increased at 1–4 hours after NGF treatment (Fig. 4E; at 1 h, 2.27 ± 0.38 fold; 2 h, 3.02 ± 0.16 fold; 4 h 3.01 ± 0.52 fold), which was followed by a steady decrease thereafter. However, in the absence of NGF, both P-Ser83-HP1γ and total HP1γ levels remained relatively constant over time (Fig. 4E; Supplementary Fig. 5). Combined, these results support the notion that the NGF-PKA pathway induces the phosphorylation of HP1γ prior to the degradation observed with our various models of neuronal cell differentiation (Fig. 2). We subsequently sought to gain insight into the function of phosphorylation of this protein during the induction of NGF-mediated neuronal cell differentiation. Toward this end, and since HP1γ is primarily known as a regulator of gene expression, we performed genome-wide transcriptomic profiling to determine the consequence of HP1γ-Ser83 phosphorylation in this process within the context of NGF-mediated PC12 cell differentiation.

### Genome-Wide Profiling Supports a Role for HP1γ In the Regulation of Gene Expression Networks Involved in NGF-mediated PC12 Differentiation

**Figure 5:**
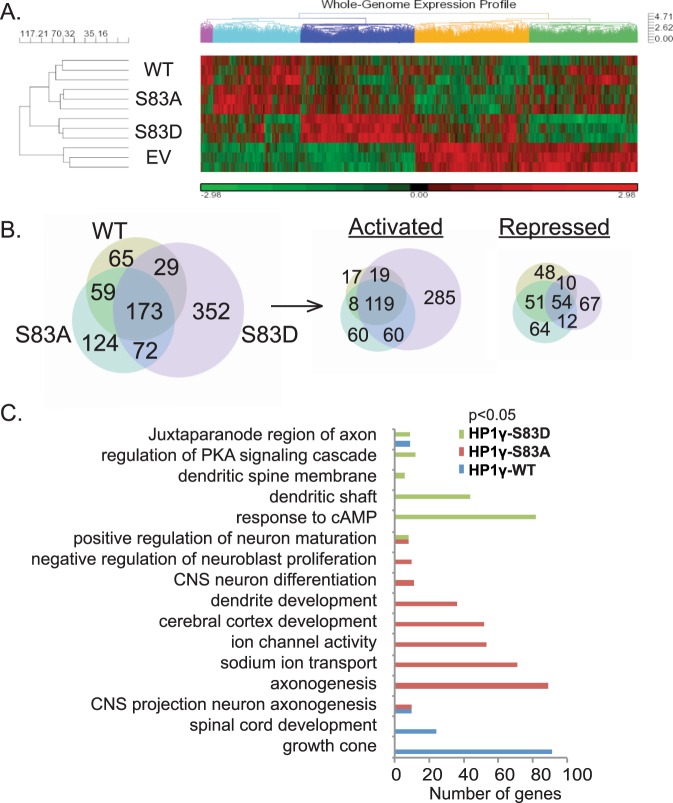
HP1γ and its S83 phosphorylation state regulate transcription of distinct gene subsets. (**A**) Heatmap of genes that are significantly altered (p > 0.05) amongst conditions of the genome-wide query from PC12 cells with control (EV) compared with HP1γ wild type (WT), HP1γ-S83A, or HP1γ-S83D overexpression. Significant (p < 0.05) genes are clustered to compare the effects of WT HP1γ and its S83 phosphorylation mutants. (**B**) The Venn diagrams display the number of genes that are significantly changed (p < 0.05, log2 fold change >1.25 or <−1.25) with overexpression of HP1γ-WT, HP1γ-S83A, or HP1γ-S83D compared to EV (left). Distribution of activated genes (middle) and repressed genes (right) compared to EV are shown separately. (**C**) GO ANOVA clusters that are significantly (p < 0.05) altered by HP1γ-WT or mutant overexpression in NGF-treated PC12 cells. Ontological classifications are shown along with the number of genes in each category altered by HP1γ-WT (blue), HP1γ-S83A (red), or HP1γ-S83D (green) overexpression.

To analyze the functional role that phosphorylation of HP1γ in response to NGF treatment may play in the context of gene expression, we performed genome-wide Affymetrix profiles of PC12 cells overexpressing wild type HP1γ, a non-phosphorylatable S83A mutant or a phosphomimetic S83D mutant. The heat map of all significantly altered genes (p < 0.05) revealed distinct clusters of genes regulated by WT-HP1γ and the S83 mutants (Fig. 5A; Supplementary Data File 1). Based on the dendogram generated by Euclidean distance calculations, wild type HP1γ and HP1γ-S83A mutant samples were more similar to each other in their effects on gene expression than with the HP1γ-S83D mutant (Fig. 5A). Using statistical and bioinformatic analyses, we identified 876 genes that differ significantly from empty vector control after wild type HP1γ or mutant overexpression (p < 0.05, log2 fold change >1.25 or <−1.25, Supplementary Data File 2). About 40% (352) of these genes were differentially regulated exclusively by HP1γ-S83D overexpression, with the majority upregulated (80%; 285 genes; Fig. 5B), further supporting a role for P-Ser83-HP1γ in inducing target gene transcription. In order to understand more specifically how HP1γ and the mutants affect NGF-mediated cell differentiation, we performed Gene Ontology (GO) ANOVA analysis with genome-wide data and investigated significantly enriched GO terms (p < 0.05) that were related to neurons and brain. Terms including ‘serotonin binding (GO:51378)’, ‘neuropeptide binding (GO:42923)’, and ‘neuropeptide Y receptor activity (GO:4983)’ were significantly enriched and downregulated in all conditions, suggesting repression of some neuronal transcriptional signatures as a result of HP1γ overexpression (Supplementary Data File 3). In addition to these commonly regulated gene sets, distinct ontological clusters related to neuronal cell differentiation were enriched with the overexpression of wild type HP1γ, HP1γ-S83A, or HP1γ-S83D (Fig. 5C; Supplementary Data File 3). Specifically, wild type HP1γ overexpression resulted in upregulation of genes related to growth cone and spinal cord development (*Erc2*, *Phox2a*, *Hoxc4*, and *Hoxb3*). HP1γ-S83D overexpression caused downregulation of the genes related to dendritic structures, such as dendritic shaft and spine (*Akap2*, *Slc12a1*, *Slc12a2* and *Lpar1*). Notably, genes involved in c-AMP and PKA signaling changed significantly with HP1γ-S83D overexpression, suggesting a role for HP1γ, and specifically modification of S83, in PKA feedback or feed-forward mechanisms. HP1γ-S83A overexpressing cells showed enriched downregulation of genes related to regulation of neuronal proliferation, differentiation, and maturation. These included gene clusters related to morphological changes, such as axonogenesis, and others related to functional maturation, like ion channel activity (*Gabrb1*, *Gabrp*, *Chrm4*, and *Chrna4*). Taken altogether, these results suggest that the phosphorylation status of HP1γ at S83 affects the expression of genes in PC12 cells that associate with neuronal cell differentiation. Next, we investigated the function in which levels of HP1γ, as well as its S83 phosphorylation, participate during the process of NGF-induced PC12 cell differentiation.

### HP1γ and its S83 Phosphorylation Behave as a Functional Barrier to NGF-Induced Neurite Formation during P12 Cell Differentiation

**Figure 6:**
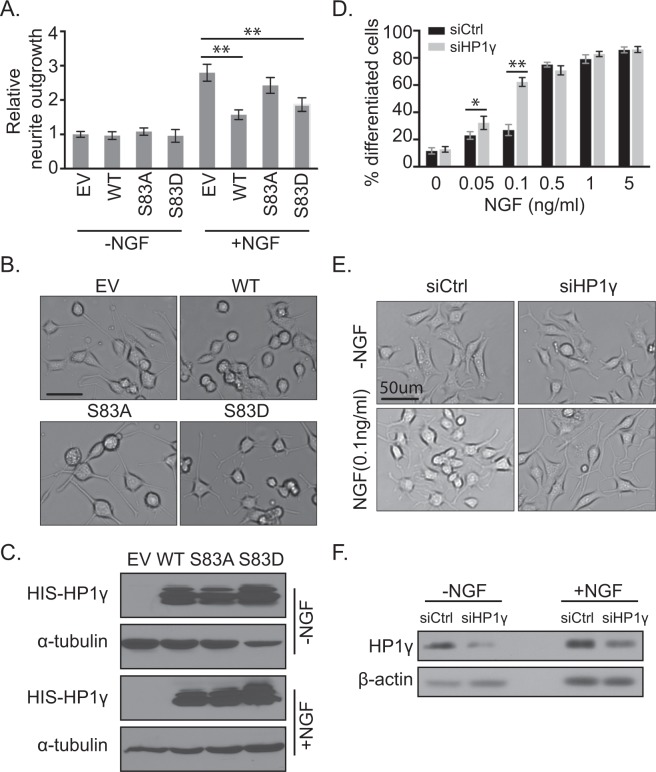
HP1γ and its Ser83 phosphorylation serve as a functional barrier of NGF-induced PC12 cell differentiation. (**A**) Quantification of neurite outgrowth is shown for wild type and mutant HP1γ overexpressing PC12 cells cultured without or with 100 ng/ml NGF for 48 hours. Neurite formation is significantly reduced with wild type HP1γ (WT) and HP1γ-S83D mutant overexpression in the presence of NGF compared to control (EV) and HP1γ-S83A mutant overexpression. (**B**) Representative images are shown from the NGF-induced conditions. (**C**) Western blot against the His-tag confirms overexpression of exogenous HP1γ proteins at 48 hours of NGF treatment. α-tubulin is used as the loading control. Full-length blots are presented in Supplementary Fig. 6B. (**D**) Quantification of PC12 cells transfected with siRNA against scrambled control (siCtrl) or HP1γ (siHP1γ) and exposed to various concentrations of NGF for 12 hours demonstrates an increased propensity for differentiation at lower NGF concentrations in siHP1γ-transfected cells. (**E**) Representative images are shown from the no NGF control and 0.1 ng/ml NGF conditions. (**F**) HP1γ knockdown by siRNA is confirmed by western blot at 12 hours of NGF treatment with β-actin as the loading control. Full-length blots are presented in Supplementary Fig. 6C. Error bars represent standard error. (*p < 0.05, **p < 0.01).

Together, our results suggested a model whereby the degradation of HP1γ, as well as its S83 phosphorylation, may play a necessary role in NGF-induced cell differentiation. To investigate further, PC12 cells were transduced with adenovirus overexpressing WT-HP1γ, the non-phosphorylatable S83A mutant or the phosphomimetic S83D mutant, and neurite formation was measured over time (48 h, Fig. 6A–C; 24 h, Supplementary Fig. 6A). By 48 hours of exogenous WT-HP1γ overexpression, antagonizing the observed reduction in endogenous HP1γ upon differentiation (Fig. 2C), NGF-induced neurite outgrowth was significantly reduced to 56.3 ± 4.9% of empty vector (EV) control levels (Fig. 6A,B; p < 0.01). Similarly, overexpression of the HP1γ-S83D mutant to specifically increase exogenous levels of HP1γ in a manner that mimics S83 phosphorylation recapitulated this decrease in neurite outgrowth (Fig. 6A,B; 66.7 ± 7.3% of control, p < 0.01). However, overexpression of a non-phosphorylatable HP1γ-S83A mutant allowed NGF-induced neurite outgrowth to occur without a significant change compared to the control cells (Fig. 6A,B; 86.8 ± 8.2%, p = 0.28), suggesting that the function of HP1γ in antagonizing neurite formation is dependent on S83 phosphorylation. In the absence of NGF, no change was observed in the amount of neurite outgrowth for any of the conditions (Fig. 6A). Expression of WT-HP1γ and the two phosphorylation mutants was controlled for relatively similar protein levels by western blot (Fig. 6C; Supplementary Fig. 6B). It is interesting to note that, distinct from the transcriptional analysis, in this functional assay HP1γ-S83D overexpression resembles WT-HP1γ overexpression while HP1γ-S83A overexpression does not differ significantly from EV control. This result demonstrates that the neurite outgrowth assay reflects only morphological aspects of cell differentiation, which can differ from the transcriptome-wide events. Conversely, cells with lower HP1γ levels, achieved by siRNA-mediated knockdown, displayed a higher propensity for dose-dependent, NGF-induced differentiation (Fig. 6D–F), in particular at low doses of NGF. Notably, whereas treatment with 0.1 ng/ml NGF for 12 hours induced neurite outgrowth in 26.9 ± 4.1% of the control cells, the outgrowth observed for cells with HP1γ knockdown was significantly higher at 62.3 ± 3.3% (Fig. 6D,E, p < 0.01). While less pronounced, this effect was also observed at an even lower dose of NGF (0.05 ng/ml) with 23.0 ± 2.3% of the control cells and 32.3 ± 4.9% of the siRNA-treated cells extending neurites (Fig. 6E, p < 0.05). Once NGF levels reached a threshold to induce most cells to undergo neurite outgrowth (0.5 ng/ml), as expected, the effect of HP1γ knockdown was no longer detectable (Fig. 6D). Knockdown of HP1γ was confirmed by western blot (Fig. 6F; Supplementary Fig. 6C). Similarly, when cells were exposed to 0.1 ng/ul of NGF for longer than 24 hours, the neurite outgrowth of control cells reached the same level as HP1γ knockdown, at which point both conditions achieved the maximum amount of neurite outgrowth (Supplementary Fig. 6D). These data demonstrate that, similar to the observed degradation of HP1γ during differentiation, siRNA-induced decrease in the level of this protein facilitates neurite outgrowth in NGF-treated PC12 cells. Thus, in summary, HP1γ is phosphorylated in response to the NGF pathway, which influences neuronal gene expression and behaves as a functional barrier during NGF-mediated PC12 cell differentiation.

## Discussion

The current study provides direct evidence supporting a role for HP1γ as a barrier in models of neuronal cell differentiation. This data is congruent with the key role of this protein in the H3K9me pathway for gene regulation. The H3K9me pathway functions as a well-known regulator of stem cell pluripotency and differentiation^25^. Furthermore, depletion of the SUV39H1 histone methyltransferase, one of the enzymes responsible for H3K9 methylation, which is bound and recruited by HP1^2^, has been found to selectively augment NGF-mediated neurite outgrowth^26^. However, the relationship of a similar function for HP1γ in neurons had remained unexplored. HP1γ is present in various sections of the brain, but decreases during brain development, indicating that its function needs to be suppressed for the normal execution of this process. We experimentally recapitulate the effects of reducing HP1γ levels on allowing neuronal cell differentiation to occur, using two cell models. We reveal that an NGF-PKA-dependent, membrane-to-nucleus signaling pathway, phosphorylates HP1γ at Ser83, a marker for the inactivation of the repressive function of this protein. Physical shifts in localization of epigenetic co-regulators, like HP1γ, provide an opportunity for genome-wide reorganization and/or re-establishment of the histone landscape, which is a critical step in differentiation^27^. Notably, we find that treatment with NGF causes a reduction of HP1γ levels in heterochromatin, which is where HP1γ performs its gene silencing function. Thus, this study defines three main post-translational mechanisms of HP1γ inactivation in the context of NGF-induced PC12 cell differentiation; namely Ser83 phosphorylation, subnuclear translocation and degradation. Since the major function of HP1γ is in the regulation of gene expression, a transcriptome-wide survey shows that overexpression of the P-HP1γ-Ser83 mimetic (HP1γ-S83D) most dramatically induced transcriptional activation of genes subsets compared to wild type or non-phosphorylatable mutant (HP1γ-S83A) overexpression. Furthermore, gene networks altered by overexpression of either wild type HP1γ or its S83 mutants include those involved in in growth cone and dendritic shaft morphogenesis, spinal cord development, neuronal, differentiation, and maturation, as well as ion channel activity.

During NGF-induced PC12 cell differentiation, P-HP1γ-Ser83 is only present during the first 4–6 hours before its subsequent degradation. In functional assays with HP1γ levels maintained at a higher level with exogenous overexpression, cells are unable to properly proceed with NGF-induced differentiation, as evidenced by a reduced amount of neurite formation. This antagonizing effect on neurite formation was also seen with HP1γ-S83D overexpression, but not with HP1γ-S83A, suggesting that S83 phosphorylation is necessary for this function. This is an intriguing point because the transcriptional signature of HP1γ-S83A overexpression is the most similar to that of the WT condition. On the other hand, in neurite outgrowth, WT and S83D overexpression show similar results, while cells with S83A overexpression extended their neurites as much as the EV control. It is important to note that S83A likely behaves as a loss of function mutant, but not as a dominant negative protein to negate the effects of endogenous HP1γ. Therefore, during our neurite outgrowth assays, providing additional amounts of HP1γ that undergo phosphorylation (WT) or mimic phosphorylation (S83D) impedes neurite formation, whereas providing non-phosphorylatable protein (S83A) has no net effect on the system, presuming the phosphorylated protein is the mediator of this effect. In regard to the transcriptional response, how phospho-mimetic and non-phosphorylatable forms of a protein work is only a biochemical approximation to the phenomena that they intend to replicate. For instance, both mutants resemble distinct biochemical states, but neither can be dynamically modified by phosphatases, in contrast to the WT protein. These data suggest that the post-translational modification status of HP1γ may be regulating different aspects of neuronal differentiation. Further studies should be conducted to further clarify the effect of non-phosphorylatable HP1γ-S83A in NGF-induced neuronal cell differentiation. Conversely, HP1γ knockdown by siRNA instigated a significant increase in neurite outgrowth in response to lower levels of NGF, below the threshold of substantial neurite outgrowth to occur under control conditions. These results are not only consistent with a role of HP1γ in neuronal cell differentiation, but also extend the mechanistic value of our study by identifying gene networks that characterize this function.

In light of the novel findings reported in this study, it becomes important to discuss their importance to the neurobiology field and potential implications for medicine. HP1γ and its associated histone methyl transferases have been linked to human diseases. Although the role of HP1γ in neurodevelopmental or neurodegenerative diseases remains unknown, our results underscore the importance of tightly regulating the levels of this protein for normal neuronal gene expression and differentiation. In addition to the abovementioned experimental evidence for a role of the histone methyltransferase SUV39H1 in neurite outgrowth, it is also relevant to discuss an additional HP1γ-associated histone methyltransferase, EHMT1/GLP, which has a significant, well-documented role in neuronal function. Indeed, mutations in EHMT1/GLP cause Kleefstra syndrome, which is a great example of an emerging group of intellectual disability disorders caused by genes encoding epigenetic regulators of neuronal gene activity^28^. Lastly, new pharmacological agents that target either HP1γ or more commonly the EHMT1/GLP-EHMT2/G9a complex have been developed^29,30^. Unfortunately, the first inhibitor to date for HP1γ has not been tested extensively and is non-specific, targeting other chromodomain-containing proteins^29^. However, based on the results described in the current study, one can expect that drugs of this type will have potentially beneficial and/or iatrogenic effects. Drugs that inhibit EHMT1/GLP are at a more advanced state of testing, and in fact, have been shown to modulate the gene expression pattern and function in several types of neurons, with beneficial effects on addictive and anxiety behaviors, as well as neuropathic hypersensitivity induced by peripheral nerve injury^31–33^. Thus, combined, this data underscores the relevance of studying HP1γ and its associated pathways for better understanding human diseases.

In conclusion, we define a role of the chromatin protein, HP1γ, in the NGF pathway response and neuronal gene expression, which also impacts differentiation of neuronal cell models. This new knowledge provided by the current study not only contributes to better understanding the role that HP1γ plays in neuronal cell differentiation, but also highlights the potential biomedical importance of this pathway.

## Materials and Methods

### Cell cultures, Reagents, and Cell treatments

PC12 cells were cultured as previously described^10^. Briefly, the cells were cultured on plates coated with poly-L-lysine and allowed to adhere overnight prior to treatment with 100 ng/ml Nerve Growth Factor (NGF; BD Bioscience), adenoviral transduction (MOI = 200), siRNA (Thermo Scientific Dharmacon), 10 μM forskolin (Calbiochem), or 5 μM KT5720 (Calbiochem), unless stated otherwise. The cells were harvested at various time points as indicated. N1E115 cells were cultured in Dulbecco’s modified Eagle’s medium high glucose with L-glutamine medium, supplemented with 10% horse serum (Invitrogen) and 0.5% penicillin-streptomycin (Sigma).

### Mouse brain samples

For western blot, brains were collected from four (2 male, 2 female) C57BL/6 mice at 6–8 weeks of age. Olfactory bulb, cortex, thalamus, hypothalamus, mid brain, hippocampus, pon and cerebellum were dissected and homogenized in Laemmli buffer. All animal protocols were approved by the Mayo Clinic Animal Care and Use Committee, and experiments were conducted in accordance with institutional guidelines and regulations.

### Immunohistochemistry

Immunohistochemistry was performed on 5 μm formalin–fixed and paraffin embedded C57BL/6 mouse brain sections (Zyagen). Sections were deparaffinized in Xylene (3 times, 5 mins each) and rehydrated by immersing the slides for two 10-minute washes in 100%, 95%, 70%, 50% ethanol followed two washes for 5 minutes in PBS/0.1% Brij-35. Antigen retrieval was performed by incubation in heated citrate buffer (10 mM) for 10 minutes. To quench endogenous peroxidase activity, the slides were incubated in 3% hydrogen peroxide solution in methanol (v/v) for 20 minutes at room temperature. Slides were permeabilized in PBS-T (0.3%, 10 min), and then endogenous antibodies were blocked by a 60-minute incubation in CAS blocking buffer. Sections were incubated with HP1γ (1:200) antibody in CAS blocking buffer overnight at 4 °C. AEC staining kit (Sigma) was used to develop, and sections were counter-stained with hematoxylin.

### Western blot

Standard western blot techniques were used to determine the levels of HP1γ and P-Ser^83^- HP1γ. Western blot samples were run on 12% SDS-PAGE gels and electroblotted onto polyvinylidene difluoride membranes (PVDF; Millipore). The membranes were blocked in 3% bovine serum albumin in TBST for 1 hour at room temperature and then incubated overnight at 4 °C with primary antibody (His-tag for recombinant HP1γ (1:1000; Santa Cruz Biotechnology), HP1γ (1:1000; Abcam), P-Ser^83^- HP1γ (1:1000; Abcam), α-tubulin (1:5000; Sigma), or β-actin (1:5000; Sigma)). HRP-conjugated anti-rabbit or mouse IgG secondary antibody (1:5000 dilution) and chemiluminescence were used to detect the protein levels.

### Isolation of Dorsal Root Ganglia (DRG)

DRG neurons were obtained from mice pups at E13^34^. Whole DRG explants were cultured on rat tail collagen-coated 35 mm plastic dishes. Nine to twelve DRG were placed on each plate at least 10 mm apart. Cultures were maintained in Eagle’s minimal essential medium containing 10% calf bovine serum, 7 mg/ml glucose, 1.2mM L-glutamine. For differentiation, whole DRG explants were treated with 10 ng/ml of NGF for 48 hours.

### qPCR

Total mRNA was extracted from cells with the RNeasy mini kit (Qiagen), and was reverse-transcribed into cDNA using the SuperScriptIII kit (Invitrogen) according to the manufacturer’s protocol. Using cDNA as the template, *Cbx3/HP1γ* transcript was amplified by qPCR and normalized to *Gapdh* (SA Biosciences).

### Immunofluorescence and pixel analysis

Immunofluorescence and confocal microscopy were performed as previously described^16^. Briefly, 0.05 × 10^6^ cells were plated on 8 well chamber slides coated with poly-L-lysine. After appropriate treatment with NGF, cells were fixed with 4% formaldehyde and permeabilized with PBS containing 0.2% triton-X. The cells were incubated with anti-HP1γ (1:200; Upstate), anti-P-Ser^83^-HP1γ (1:200; Abcam) or anti-H3K9me3 (1:100; Millipore) primary antibodies for 2 hours at 37 °C and then with fluorescein secondary antibodies for 1 hour at 37 °C. Direct labeling (ThermoFisher Scientific) was used for pre-labeling H3K9me3 antibody when needed. Cell nuclei were counterstained with DAPI-containing mounting media (Vector Labs). Fluorescence signals were observed with 488- and 568 nm excitation wavelengths on Zeiss LSM-710 confocal laser scanning microscope. Zen 2009 software was used for pixel analysis. The numbers of pixels activated by each channel were counted and the percentage co-localization between HP1γ and H3K9me3 was calculated in 200 or more cells per condition.

### Neurite outgrowth

On poly-L-lysine-coated 6-well plates, 0.5 × 10^6^ PC12 cells were plated and allowed to adhere overnight. 12 hours prior to NGF treatment, cells were treated with siRNA or adenovirus (MOI = 200). Various concentrations of NGF were added to complete media, and light microscopic images were acquired. Images of random fields were taken, and neurite outgrowth was assessed using stereology. A grid was overlaid to the images to count the total number of intersections between neurites and the grid and then divided by the number of somites. The ‘relative neurite outgrowth’ was calculated by comparing the neurite/somite ratio to EV, no NGF condition. The ‘% differentiated cells’ was calculated by the number of cells with neurites longer than twice the soma length over the total number of cells. Based on the time course of neurite outgrowth, we selected time points that showed the most pronounced effects of the knockdown (12 h; Supplementary Fig. 6D) and overexpression (48 h; Fig. 6A). Overexpression and knockdown of HP1γ were confirmed by western blot. At least 400 cells were examined from ten to twenty fields for each condition.

### Recombinant adenovirus and siRNA

Epitope-tagged (6XHis-Xpress™) HP1γ-wild type (WT), HP1γ-S83A, and HP1γ-S83D, as well as empty vector (EV, Ad5CMV), were generated as previously described^18^. Rat HP1γ (Cbx3) ON-TARGETplus SMARTpool siRNA was purchased from Thermo Scientific Dharmacon and used as suggested in the manufacturer’s protocol.

### GST Fusion Protein Purification and *in vitro* Phosphorylation Assays

GST fusion protein purification was performed as previously described^16^. For PKA *in vitro* kinase assay, wild type HP1 fusion proteins (10 µg) were incubated with recombinant kinases (Millipore) and 10 mM ATP (Sigma) for 10 min at 30 °C in buffer containing 25 mM Hepes-KOH at pH 7.5, 30 mM NaCl, 10 mM MgCl_2_, and 0.5 mM DTT. Kinase reactions were terminated by the addition of SDS loading dye, then resolved by western blot as described above.

### Gene Expression Profiling-Microarray Analysis

PC12 cells were transduced with adenovirus (MOI = 200) containing the control empty vector (EV), HP1γ-WT, HP1γ-S83A, or HP1γ-S83D constructs. Six hours later, cells were treated with 100 ng/ml NGF for 48 hours and then were lysed for total mRNA extraction. Global gene expression profiling was carried out at the Microarrays Facility in the Research Center of Laval University CRCHUL utilizing Affymetrix GeneChip^®^ Rat Gene 2.0 ST Array. Intensity files were generated by Affymetrix GCS 3000 7 G and the Gene-Chip Operating Software. Data analysis, background subtraction and intensity normalization were performed using Robust Multiarray Analysis^35^. Differentially expressed genes and the false discovery rate were estimated by t-test (>0.005) and corrected using Bayes approach^36,37^. Data analysis, hierarchical clustering, and ontology were performed with the OneChanelGUI to extend affylmGUI graphical interface capabilities^38^, and Partek Genomics Suite, version 6.5 (Partek Inc.) with ANOVA analysis. Probes with p-value < 0.05 and fold change ±2.2 among HP1γ-WT vs empty vector (EV), HP1γ-S83A vs EV, and HP1γ-S83D vs EV were selected for further analysis. For Gene Ontology (GO) ANOVA a minimum threshold of 3 genes and a p < 0.05 was used to identify significant functional groups. To validate the Affymetrix microarray, targets with significant alteration (p < 0.05) were compared to the real-time data using an arbitrary cutoff of ±2.2 fold change compared to EV control.

## Electronic supplementary material

Supplementary Information

Supplementary Data File 1

Supplementary Data File 2

Supplementary Data File 3
