# Supplementary material for: Mechanisms Underlying the Regulation of HP1γ by the NGF-PKA Signaling Pathway
Source: Sci Rep. 2018 Oct 10;8:15077. doi: 10.1038/s41598-018-33475-y (PMC6180112; doi:10.1038/s41598-018-33475-y)
Supplement: Supplementary file 1 — Supplementary Information [file 41598_2018_33475_MOESM1_ESM.pdf]

Supplementary Information

Mechanisms Underlying the Regulation of HP1γ by the NGF-PKA Signaling Pathway

Seungmae Seo[1], Angela Mathison[2], Adrienne Grzenda[3], Jewel Podratz[4], Ezequiel Calvo[5], Stephen Brimijoin[6], Anthony Windebank[4], Juan Iovanna[7], Gwen Lomberk[8,9*] and Raul Urrutia[2,8*]

[1] Lieber Institute for Brain Development, Baltimore, MD, USA;
[2]Genomic Sciences and Precision Medicine Center (GSPMC), Medical College of Wisconsin, Milwaukee, WI, USA;
[3]University of California, Los Angeles, Psychiatry Residency Program, Los Angeles, CA, USA;
[4]Department of Neuroscience, Mayo Clinic, Rochester, MN, USA;
[5]Centre Génomique du Centre de Recherche du CHUL Research Center, Ville de Québec, Quebec, Canada;
[6]Department of Pharmacology and Experimental Therapeutics, Mayo Clinic, Rochester, MN, USA;
[7]Centre de Recherche en Cancérologie de Marseille (CRCM), INSERM U1068, CNRS UMR 7258, Institut Paoli-Calmettes, Aix Marseille Université, Marseille, France
[8]Division of Research, Department of Surgery, Medical College of Wisconsin, Milwaukee, WI, USA;
[9]Department of Pharmacology and Toxicology, Medical College of Wisconsin, Milwaukee, WI, USA

[*]Co-corresponding authors: glomberk@mcw.edu or rurrutia@mcw.edu

Supplementary Figure 1

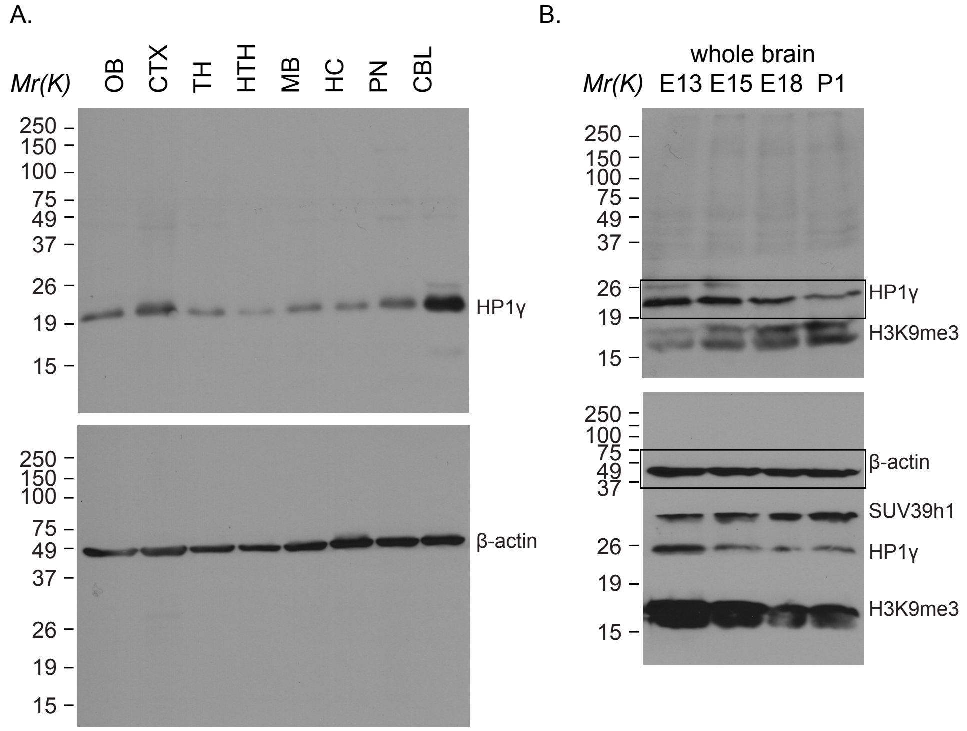

Supplementary Figure 1. Full-length blot images from Figure 1B and 1C: A. Western blot images of HP1γ (top) and β-actin (bottom) protein levels in dissected mouse brain areas (ob: olfactory bulb, ctx: cortex, th: thalamus, hth: hypothalamus, mb: mid brain, hc: hippocampus, pn:pons, cbl: cerebellum). The cropped images are shown in **Figure 1B**. **B.** Western blot images of HP1γ (top, bottom) and β-actin (bottom) in mouse whole brain samples from embryonic (E) days 13, 15, 18 and post-natal (P) day 1. Blots were also simultaneously probed for H3K9me3 (top, bottom) and SUV39H1 (bottom), which were not followed for this study. The cropped images are shown in **Figure 1C**.

Supplementary Figure 2

A. BrainSpan Dataset:

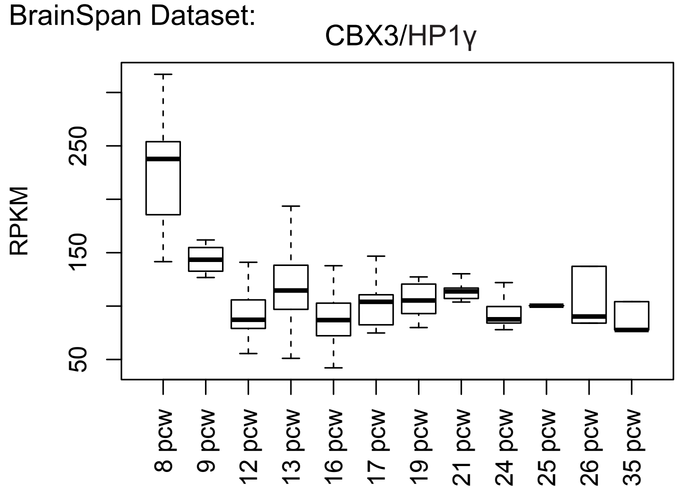

B. Cortecon Dataset:

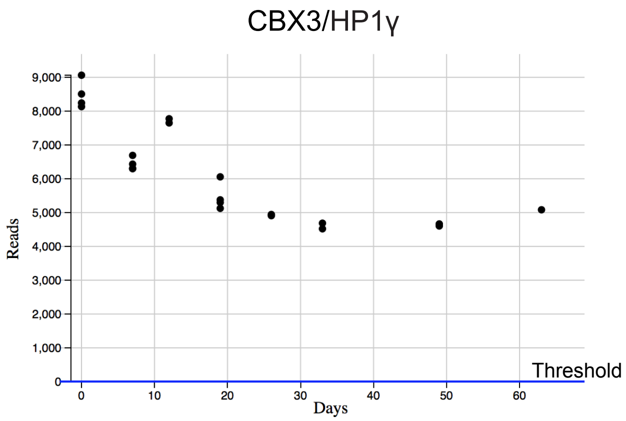

Supplementary Figure 2. CBX3 expression in brain development and differentiation from public datasets: A. RNA seq data from the BrainSpan dataset of developing human brain shows decrease of HP1γ (CBX3) mRNA levels across time in gestation. (pcw: postconception weeks) **B.** Data from Cortecon shows a decrease of HP1γ (CBX3) mRNA levels across time as iPSC differentiate into neurons.

Supplementary Figure 3

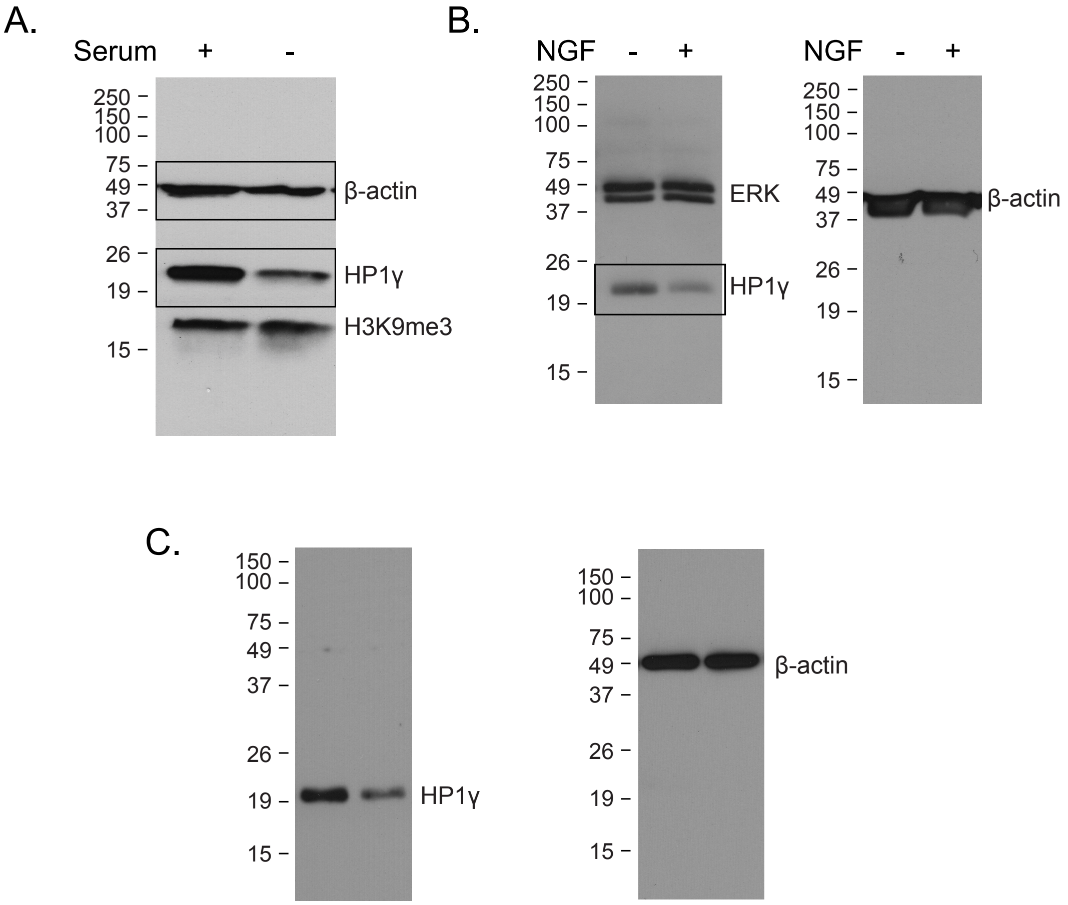

Supplementary Figure 3. Full-length blot images from Figure 2A and 2C: A. Western blot image of HP1γ and β-actin protein levels in the N1E115 cell line under control and serum starved conditions. Blot was also simultaneously probed for H3K9me3 (bottom bands), as an additional control, but not shown in the final figure. The cropped images are shown in **Figure 2A**. **B.** Western blot images of HP1γ (left) and β-actin (right) in PC12 cells under control conditions or differentiated with 72 hours of 100ng/ml NGF treatment. Right blot was also simultaneously probed for ERK (top bands), as an additional control, but not shown in the final figure. The cropped images are shown in **Figure 2C**. **C.** Western blot images of HP1γ and β-actin protein levels in whole DRG from E13 differentiated with 10ng/ml NGF treatment for 48 hours. The cropped images are shown in **Figure 2E**.

Supplementary Figure 4

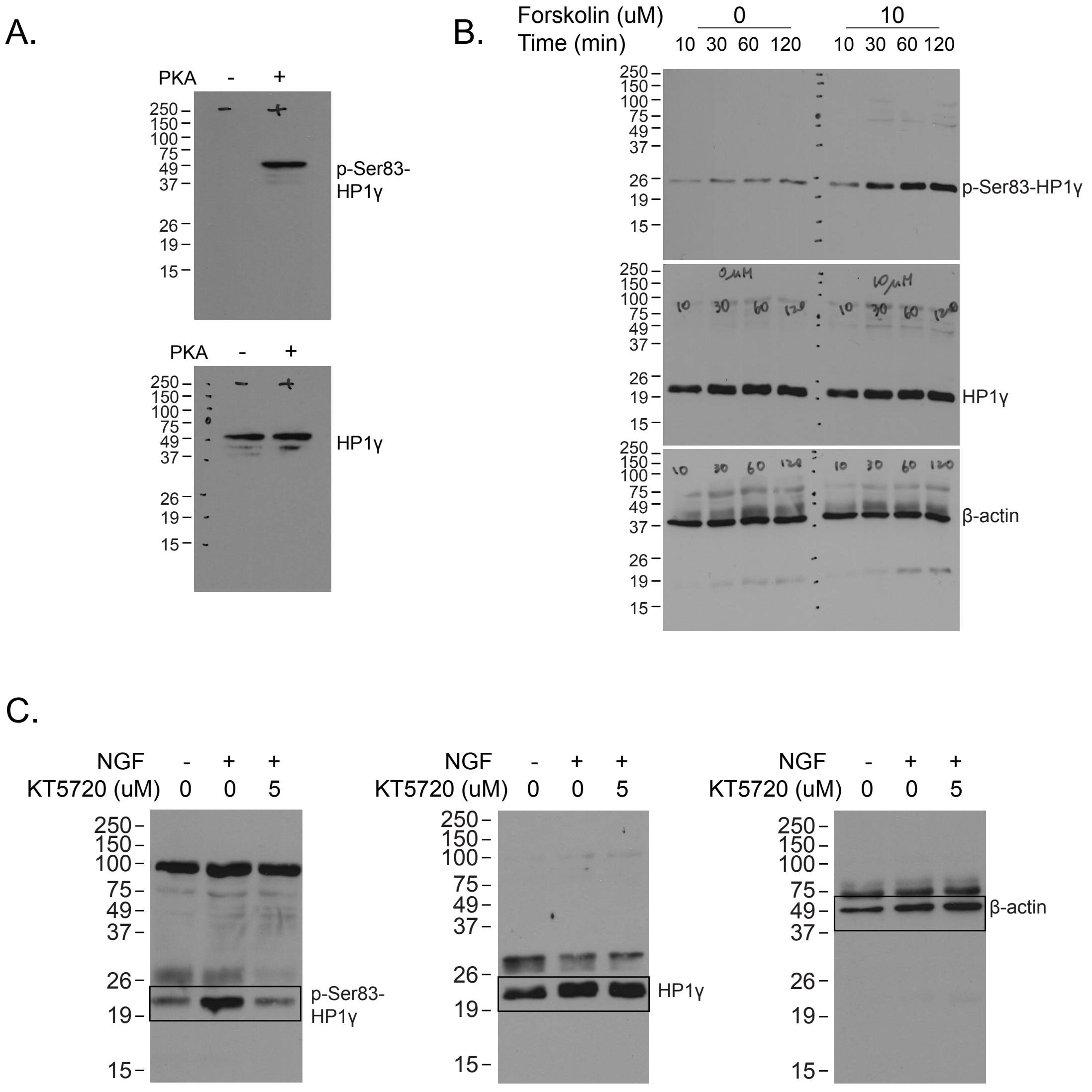

Supplementary Figure 4. Full-length blot images from Figure 4B, 4C and 4D: A. Western blot image of purified GST-HP1γ incubated with PKA *in vitro* probed for p-Ser83-HP1γ (top) and total HP1γ (bottom). GST-HP1γ fusion protein has a MW of approximately 50kD. The cropped images are shown in **Figure 4B**. **B.** Western blot images of p-Ser83-HP1γ (top), total HP1γ (middle) and β-actin (bottom) in PC12 cells after forskolin (10μM) treatment for the indicated time points. The cropped images are shown in **Figure 4C**. **C.** Western blot images of p-Ser83-HP1γ (left), total HP1γ (middle) and β-actin (right) in PC12 cells under control (-NGF) or differentiating conditions after 72 hours of either NGF (100ng/ml; +NGF) alone or NGF (100ng/ml) plus KT5720 treatment (5uM). The cropped images are shown in **Figure 4D**.

Supplementary Figure 5

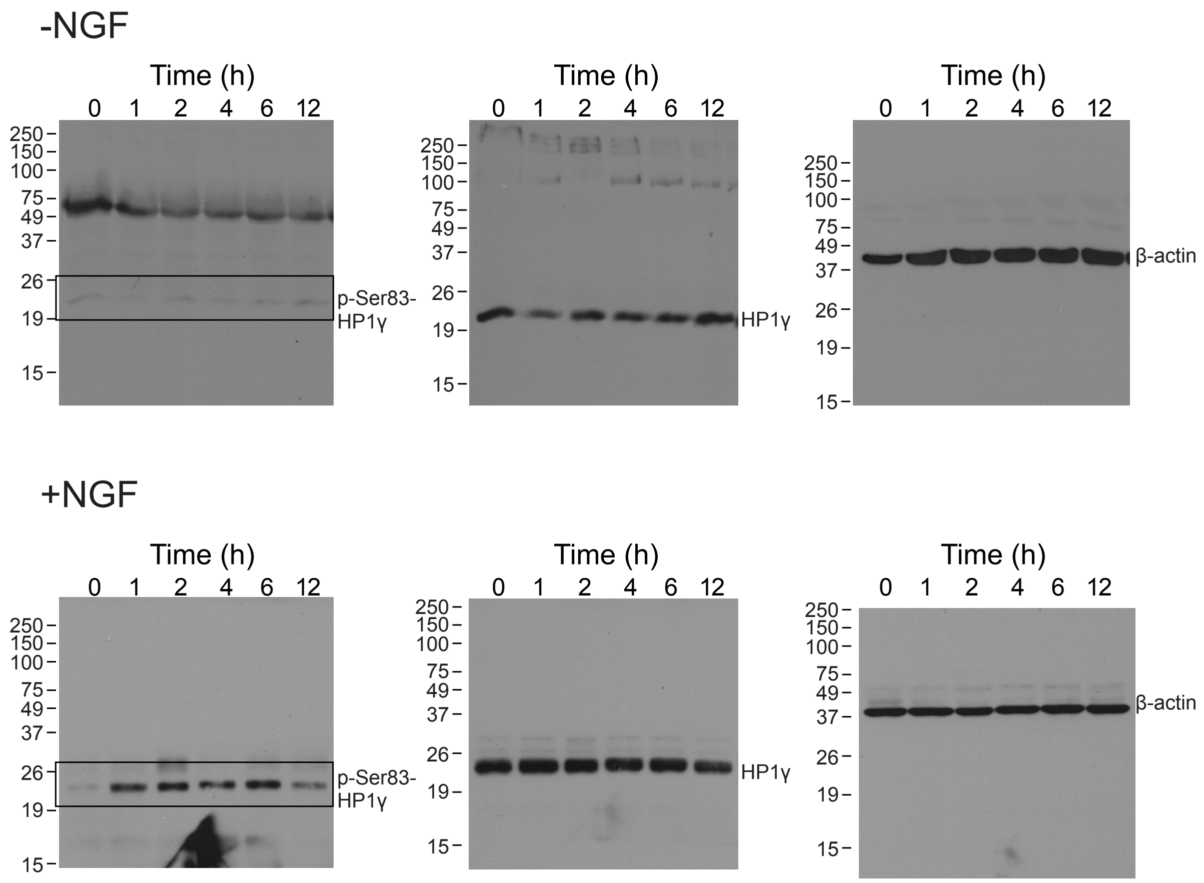

Supplementary Figure 5. Full-length blot images from Figure 4E: Western blot images of p-Ser83-HP1γ (left panels), total HP1γ (middle panels) and β-actin (right panels) in PC12 cells under control conditions (-NGF, top blots) or with 100ng/ml of NGF (+NGF, bottom blots) for the indicated time points. The cropped images are shown in **Figure 4E**. Note that p-Ser83-HP1γ is not detected under control (-NGF conditions).

Supplementary Figure 6

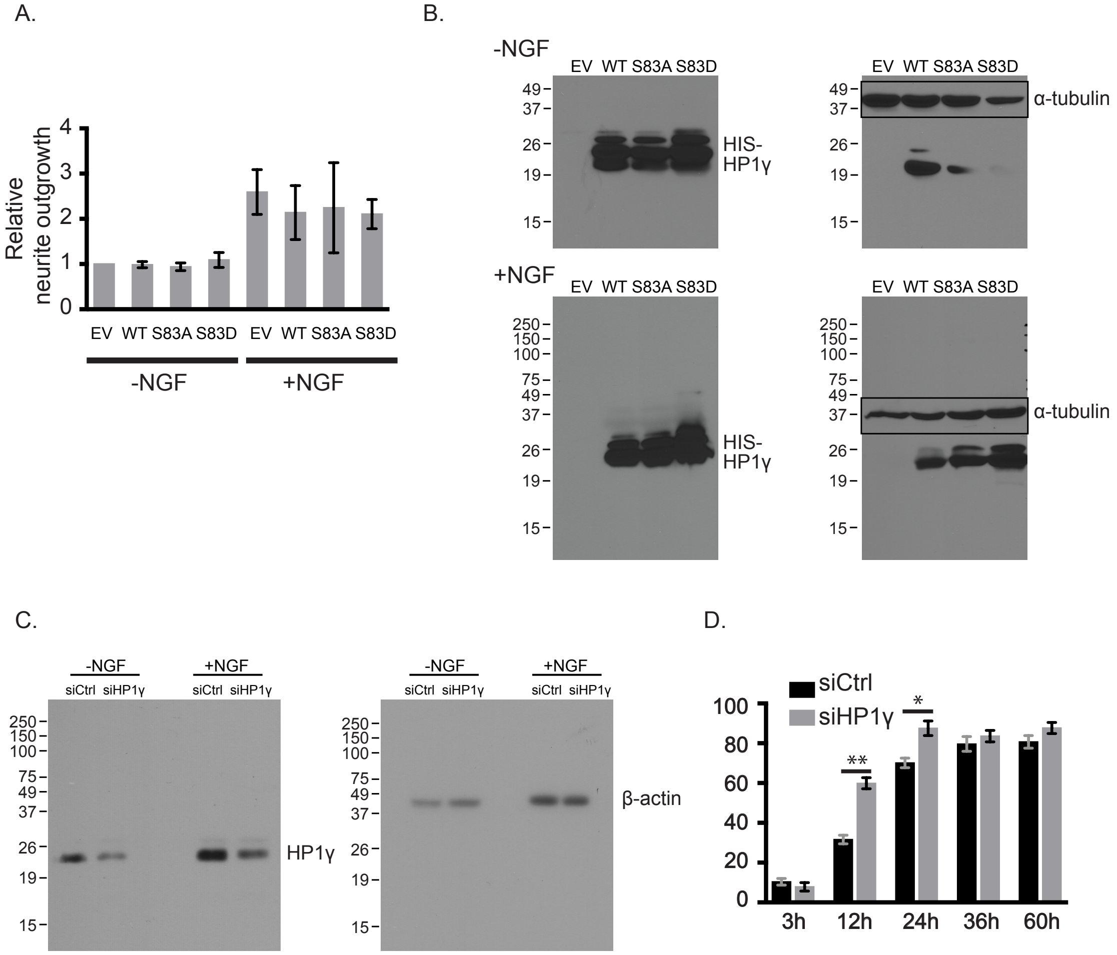

Supplementary Figure 6. Additional time points for neurite formation and full-length blot images from Figure 6C and 6F: A. Quantification of neurite outgrowth is shown for wild type and mutant HP1γ overexpressing PC12 cells cultured without or with 100ng/ml NGF for 24 hours. At this time point, there was no significant difference in neurite outgrowth with wild type HP1γ (WT) and HP1γ-S83D mutant overexpression in the presence of NGF compared to control (EV) and HP1γ-S83A mutant overexpression. **B.** Western blot image of His-tagged HP1γ WT, S83A and S83D probed for His-tag (left) and re-probed with α-tubulin (right). EV lysates were from empty vector control transduced cells. Lysates were collected at the 48hr time point. The cropped images are shown in **Figure 6C**. **C.** Western blot images of HP1γ knockdown by siRNA (siHP1γ) probed for HP1γ (left) and β-actin (right) in PC12 cells without or with NGF. Scrambled siRNA control lysates are indicated as siCtrl. Lysates were collected at the 12hr time point. The cropped images are shown in **Figure 6F**. **D.** Time course quantification of PC12 cells transfected with siRNA against scrambled control (siCtrl) or HP1γ (siHP1γ), exposed to 0.1ng/ml concentrations of NGF demonstrate that the effect of HP1γ is most prominent at 12h. (* $p<0.05$, ** $p<0.01$)
